# Cross-Sectional and Longitudinal Associations Among Weight Stigma, Psychological Distress, and Eating Behaviors in Youth with Obesity: A Clinical Sample

**DOI:** 10.3390/medicina61030466

**Published:** 2025-03-07

**Authors:** Wee Shen Khoo, Ying-Chu Chen, Yen-Yin Chou, Yu-Wen Pan, Yun-Han Weng, Meng-Che Tsai

**Affiliations:** 1 Department of Pediatrics, National Cheng Kung University Hospital, College of Medicine, National Cheng Kung University, Tainan 704302, Taiwan; ganzykhoo89@gmail.com (W.S.K.); yenyin@mail.ncku.edu.tw (Y.-Y.C.); panyuwen0527@hotmail.com (Y.-W.P.); 10209023@gs.ncku.edu.tw (Y.-H.W.); 2 Department of Nursing, National Cheng Kung University Hospital, Collage of Medicine, National Cheng Kung University, Tainan 704302, Taiwan; t26071076@gs.ncku.edu.tw; 3 Department of Genomic Medicine, National Cheng Kung University Hospital, College of Medicine, National Cheng Kung University, Tainan 704302, Taiwan; 4 Department of Medical Humanities and Social Medicine, School of Medicine, National Cheng Kung University, Tainan 704302, Taiwan; 5 Institute of Clinical Medicine, College of Medicine, National Cheng Kung University, No. 1 University Road, Tainan 70101, Taiwan

**Keywords:** obesity, youth, weight stigma, depression, anxiety, eating behaviors, body mass index

## Abstract

*Background and Objectives*: Obesity in youth is a growing public health concern, placing them at higher risk for adverse physical and psychological outcomes. Understanding the predictors that affect weight management, particularly the role of internalized weight stigma, psychosocial factors, and eating behaviors, is essential for developing an effective intervention at longitudinal follow-up. *Materials and Methods*: We enrolled 102 youths with obesity aged 10 to 18 years old from clinical settings. Baseline demographic data, psychosocial measures, including the Weight Self-Stigma Questionnaire (WSSQ) and Hospital Anxiety and Depression Scale (HADS), and eating behavior scales, such as the Three-Factor Eating Questionnaire (TFEQ-R21) and eating disorder as Sick, Control, One, Fat, Food questionnaire (SCOFF), were collected in the first visit. We conducted a study with both cross-sectional and longitudinal components. Correlational bivariate analysis was conducted to explore relationships between key variables. The factors affecting BMI changes were investigated using generalized estimating equations (GEEs) as part of a longitudinal analysis. *Results*: The mean age of participants was 13.22 years and 63.7% were male. Bivariate correlation analysis revealed positive relationships between initial BMI Z-scores and WSSQ scores (r = 0.196, *p* < 0.05). In bivariate analysis, a negative correlation was found between the difference in BMI Z-scores and visit number (r = −0.428, *p* < 0.01). GEE analysis demonstrated that initial BMI Z-scores (coefficient = 1.342, *p* < 0.001) and anxiety (coefficient = 0.050, *p* < 0.001) were significant positive predictors of BMI Z-scores, while depression was negatively associated (coefficient = −0.081, *p* < 0.001). Excluding the TFEQ subscales, SCOFF improved the model’s QIC and highlighted WSSQ as a significant, albeit weak, predictor (*p* = 0.615 in the full model versus *p* < 0.05 in the reduced model). *Conclusions*: Psychosocial factors, particularly anxiety and weight stigma, are associated with elevated BMI Z-scores in youth affected by obesity in this study. The baseline age, BMI Z-score, internalized weight stigma, and psychological stress influenced the body weight trajectory over time. Frequent clinical follow-ups contribute to improved BMI outcomes. Future research may examine the efficacy of weight management by reducing weight stigma and psychological distress along with the outpatient care of obesity.

## 1. Introduction

The prevalence of obesity among children and adolescents, including those in Taiwan, has significantly increased in recent years, raising considerable public health concerns [1,2,3]. The overall prevalence of overweight and obesity among Taiwanese youth is 28.2% [1]. When obesity coexists with physical comorbidities—such as diabetes, hypertension, and dyslipidemia—management becomes incredibly challenging [4,5,6,7]. Beyond this, obesity is also associated with psychosocial burdens in youths, who often struggle with depression, anxiety, and body image dissatisfaction. In the following paragraph, we discuss how such distress may contribute to unhealthy eating behaviors [8,9].

A key contributor to these psychosocial burdens is weight stigma, defined as the attribution of negative beliefs, discrimination, and stereotypes toward individuals perceived as overweight or obese [10,11]. An alarming fact is that between 13 and 32% of youth have experienced discrimination based on their weight status [11]. These stigmatizing experiences become internalized as internalized weight stigma (IWS), characterized by self-devaluation, low self-esteem, and fear of enacted bias. Research indicates that youth experiencing weight-related stigma are at an elevated risk for psychological stress, including increased anxiety, depression, and diminished self-esteem [6,10,12]. These psychological stressors can drive maladaptive eating behaviors, such as emotional or uncontrolled eating and cognitive restraint, potentially increasing the risk of eating disorders (EDs) [5,13,14,15].

The prevalence of EDs was higher in youths with obesity when compared to the healthy population [16]. Evidence suggests that individuals with EDs often experience lifetime obesity, particularly among females [17]. Moreover, unhealthy eating behaviors such as emotional eating and overeating contribute to lifestyle-related risk factors for obesity [15,18].

Beyond these health burdens, youth with obesity often face weight-related teasing from peers, parents, and teachers in various contexts [10,11,19]. These stigmatizing environments can discourage individuals with obesity from seeking healthcare services [20,21] and have been linked to attrition in clinical follow-up [22]. Although several cross-sectional studies have documented associations among weight stigma, psychological distress, and disordered eating behaviors in youth with obesity [6,10,18,23,24], such study designs do not capture how these factors affect youth longitudinally without medical intervention.

The longitudinal effects of weight management in youth with obesity are influenced by factors such as IWS, psychological stress, unhealthy eating behaviors, and EDs, particularly in community settings [8,25,26].

Psychosocial factors may contribute to the weight status of youth with obesity. As mentioned in the previous literature, we hypothesize that higher levels of internalized weight stigma, anxiety, and depression will be correlated with higher BMI z-scores at baseline. In addition, higher internalized weight stigma and psychological distress at baseline may predict less favorable BMI-z change over the follow-up period.

## 2. Materials and Methods

### 2.1. Participants and Outpatient Follow-Up

From 2017 to 2023, we recruited 102 Taiwanese youths (10–18 years of age) with obesity, defined as a body mass index (BMI) at or above the 95th percentile for age and sex. All participants had obesity with no additional comorbidities. At the initial screening, those presenting with underlying metabolic syndrome, genetic disorders, or oncological diseases were excluded. After both parental consent and participant assent were obtained, each participant completed a self-administered questionnaire assessing internalized weight stigma, psychological stress, eating behaviors, and eating disorders. This study was approved by the Institutional Review Board of the National Cheng Kung University Hospital (B-BR-105-062).

According to the Taiwanese guideline on pediatric obesity management [27], participants were scheduled for outpatient follow-up visits every 3 months. Each visit included office consultations and weight control counseling, emphasizing comprehensive lifestyle modifications—such as dietary guidance and physical activity planning—without pharmacological interventions. Psychological support was also provided when needed.

For the purposes of this study, follow-up frequency was defined as the total number of outpatient visits over the study period, whereas the visit interval was calculated as the time between two recorded visits (in years). Overall, the mean duration of outpatient follow-up was 2.02 years.

### 2.2. Arthrometric Assessments

Height and weight were measured using a calibrated stadiometer and digital scale during the outpatient visit. The body weight was recorded following the standard method for calculating Taiwanese body mass index [28], but the BMI cannot directly represent the actual weight in different ages, so we converted BMI to the BMI Z-score (BMI-Z) to assess the longitudinal change among the youth with obesity. We also collected the difference in BMI-Z (DBMI-Z) between the first and the last visit in the outpatient department.

### 2.3. Instruments

- Internalized Weight Stigma

This involved assessing the degree of internalized weight stigma to which a participant experiences weight-related discrimination and self-devaluation using a validated self-report questionnaire, such as the Weight Self Stigma Questionnaire (WSSQ) [23,29]. The Taiwan version of WSSQ contains 12 items subdivided into 2 subscales, including self-devaluation and fear of enacted stigma, with total scores ranging from 12 to 60. Higher scores indicate greater self-stigma. Cronbach’s alpha values were 0.801 and 0.892, respectively [14]. Several studies have demonstrated a correlation between the WSSQ and measures of psychological distress, weight status, and eating attitudes in subjects with overweight and obesity [14].

- Psychological stress

The level of depression and anxiety were measured using the Hospital Anxiety Depression Score (HADS) for the youth in clinical and community settings [30,31], consisting of 14 items using a 4-point scale, ranging from 0 to 3 (0: absence of symptoms, 3: severe symptoms), and containing 2 subscales, depression and anxiety, each with 7 items [32,33]. There are 3 cutoff levels: a score between 8 and 10 indicates a mild case, a score between 11 and 14 represents a moderate case, and 15 or above marks a severe case [34]. Cronbach’s alpha of HADS was 0.813 for anxiety and 0.692 for depression [35].

- Eating behaviors

We assessed participants’ eating behaviors using the Taiwanese version of the Three-Factor Eating Questionnaire in 21 statements (TFEQ-R21) [9]. The self-administered questionnaire encompasses 3 subdomains—Emotional Eating (EE), Uncontrolled Eating (UE), and Cognitive Restraint (CR) [36]. This instrument employs a four-point Likert scale, allowing participants to indicate the extent to which each statement reflects their own eating attitudes. Previous research has demonstrated the reliability of the TFEQ-R21 in evaluating eating behaviors among youth; Cronbach’s alpha was 0.84 for the UE domain, 0.91 for the EE domain, and 0.79 for the CR [9,23].

- Eating disorders

The SCOFF comprises 5 dichotomous items addressing behaviors and attitudes related to disordered eating, including purging, binge eating, significant weight loss, body dissatisfaction, and a feeling of being controlled by food [37]. Based on prior validation studies, a score of 2 or more (yes to 2 or more of 5 questions) is proposed to detect the potential eating disorder [38]. The Mandarin version of the SCOFF we employed is culturally and linguistically appropriate in the Taiwanese social context; Cronbach’s alpha was 0.519 in a previous study [9,39].

### 2.4. Statistical Analysis

We first conducted descriptive analyses to summarize participant characteristics and distributional properties of the key variables. We also explored initial associations between variables such as age, gender, BMI, BMI-Z, DBMI-Z, visit number, visit interval, IWS measurement, psychological stress levels, eating behavior, and eating disorders, measured by employing bivariate correlations (Pearson’s correlation).

To model the longitudinal data, we utilized generalized estimating equations (GEEs) which appropriately accounted for the longitudinal data of BMI-Z over time. GEE models offer a distinct advantage over traditional random-effects approaches, as they yield consistent parameter estimates, even considering within-subject correlation, thus helping us to analyze the repeated measure of body weight [40]. The age, gender, and baseline BMI were adjusted as covariates. All statistical analyses were performed using SPSS version 27.0. A *p*-values of <0.05 was considered statistically significant.

## 3. Results

The study included a total of 102 participants, with an average age of 13.22 years (SD = 2.42), 63.7% of whom were male (as seen in Table 1). The initial body mass index (IBMI) had a mean of 30.83 (SD = 4.88) and the initial BMI Z-score (IBMI-Z) averaged 2.14 (SD = 0.35). Over the study period, the mean DBMI-Z was −0.16 (SD = 0.51) (presented in Table 1). Participants attended a mean of 7.74 visits (SD = 8.62), with an average visit interval of 0.33 years (SD = 0.39).

As shown in the correlation matrix (Table 1), gender was negatively associated with visit number (r = −0.212, *p* < 0.05) and cognitive restraint (TFEQ-R21_CR; r = −0.204, *p* < 0.05). In other words, female participants tended to have a slightly higher number of follow-up visits and reported higher levels of cognitive restraint relative to male participants. Meanwhile, gender was not significantly related to initial BMI (IBMI; r = 0.015, *p* > 0.05), BMI z-score at baseline (IBMI-Z; r = −0.185, *p* > 0.05), or changes in BMI z-score across visits (DBMI-Z; r = −0.156, *p* > 0.05).

We note that 46.46% of participants scored ≥ 2 on the SCOFF, indicating possible disordered eating. The average HADS_Anxiety was 11.69 ± 3.69, indicating a moderate level of anxiety. The average HADS_Depression was 8.20 ± 3.02, which showed a mild level of depression. Other mean scale scores appear in Table 1 but are not discussed in detail here.

Bivariate correlations between key variables were analyzed, as demonstrated in Table 1. Significant positive correlations were observed between IBMI-Z and WSSQ (r = 0.196, *p* < 0.05), WSSQ and EE (r = 0.262, *p* < 0.01), WSSQ and UE (r = 0.323, *p* < 0.01), and SCOFF and WSSQ (r = 0.426, *p* < 0.01). HADS_Depression was positively correlated with SCOFF (r = 0.269, *p* < 0.01), as was HADS_Anxiety (r = 0.195, *p* < 0.05). Among the TFEQ-R21 subscales, UE was positively correlated with EE (r = 0.507, *p* < 0.01). The visit number was negatively associated with DBMI-Z (r = −0.428, *p* < 0.01).

However, certain variables showed non-significant correlations. For instance, DBMI-Z was not significantly correlated with WSSQ (r = 0.138, *p* > 0.05), HADS_Anxiety (r = 0.135, *p* > 0.05), or HADS_Depression (r = −0.116, *p* > 0.05).

The GEE analysis examined the association between BMI Z-score and key demographics, IWS, psychological stress, eating behaviors, and eating disorders, as shown in Table 2. The results demonstrated that a higher initial BMI Z-score (IBMI-Z) was significantly associated with an increased BMI Z-score (coefficient = 1.342, 95% CI: 1.210 to 1.473, *p* < 0.001). Visit numbers showed a negative association with BMI Z-score (coefficient = −0.028, 95% CI: −0.033 to −0.023, *p* < 0.001), suggesting that more frequent visits were associated with a reduction in BMI Z-score.

Psychological factors significantly influenced BMI Z-scores. Higher HADS_Anxiety scores were positively associated with BMI Z-score (coefficient = 0.050, 95% CI: 0.033 to 0.068, *p* < 0.001), while higher HADS_Depression scores were inversely associated (coefficient = −0.081, 95% CI: −0.094 to −0.067, *p* < 0.001).

Age and visit interval time were not significant predictors of BMI Z-score, with coefficients of 0.016 (95% CI: −0.002 to 0.035, *p* = 0.083) and 0.008 (95% CI: −0.086 to 0.101, *p* = 0.872), respectively. Weight-related stigma, as measured by the WSSQ, did not demonstrate a significant association (coefficient = 0.002, 95% CI: −0.006 to 0.009, *p* = 0.615).

The exclusion of these variables improved the model fit, as reflected by a lower quasi-likelihood under the independence model criterion (QIC). By excluding the non-contributing variables, such as TFEQ subscales and SCOFF, the model’s QIC was reduced (from 251.40 decreased to 216.75). As can be seen in Table 3, the final model enhanced its fit and allowed WSSQ to emerge as a statistically significant predictor (coefficient = 0.006, *p* < 0.05) in the simplified mode.

## 4. Discussion

The study was built on identifying the correlations among the variables and underscoring predictive factors influencing the longitudinal change in BMI Z-scores. Firstly, the positive coefficient between IBMI-Z and subsequent BMI Z-scores suggests that baseline weight status may influence the outcome and adherence to weight management [41,42,43]. This underscores the importance of early identification and targeted intervention for youths with obesity [43], as they may face greater challenges in achieving weight reduction goals.

The data also demonstrate that more frequent clinic visits were associated with lower BMI Z-scores, indicating the value of consistent engagement with weight management services. Frequent and consistent appointments may provide increased support and counseling and motivate lifestyle modification—all of which may be particularly crucial for youths with obesity who have not received the medication intervention. The guideline highlighted the intensity of the clinical contact, and counseling demonstrated the reduction in BMI [44,45]. This relationship is likely bi-directional: adolescents who experience initial success may be more motivated to return, while additional visits provide ongoing support and enhance adherence, further promoting success.

The finding that female participants had more frequent visits and exhibited higher cognitive restraint than their male counterparts is consistent with the literature suggesting that females may be more proactive in health monitoring and more inclined toward dietary self-regulation [46,47]. This difference in health-seeking behavior and restraint could reflect social or cultural expectations placed on females regarding body image and diet-related cognitions.

Nevertheless, the non-significant effect of gender on BMI changes within our GEE model suggests that these gender-based differences in visit frequency and cognitive restraint did not translate into marked differences in longitudinal weight outcomes. Other factors such as developmental stage, psychosocial influences, and contextual variables (e.g., family environment, family socioeconomic status, lifestyle practices) may override or mediate the potential impact of gender on weight trajectories.

The association between obesity and psychological distress (like anxiety and depression) is consistent with the recent literature [6,48,49]. The longitudinal study demonstrated that anxiety and depression increased the risk of development of obesity [50,51]. Exploration of the mediated effect revealed that a person with obesity and psychological distress is more likely to develop disordered eating behaviors as the strategy of emotional regulation [9,49,52,53].

Notably, moderate severity of anxiety was associated with increased BMI Z-scores, while mild severity of depression was linked to decreased BMI Z-scores in our study, which is different from other studies. These divergent patterns may reflect differences in coping mechanisms—such as emotional eating and uncontrolled eating in anxiety [53]—or dietary restraint in youths with obesity who had higher levels of depression [54]. Such findings indicate that mental health status can powerfully shape weight-related behaviors and outcomes among youths [55]. These results emphasize the importance of screening for anxiety and depression within weight management programs and tailoring treatment plans to address the specific psychological profiles of adolescents.

Additionally, we found that the correlation linked participants’ weight status to a higher level of IWS. This suggests that youths with obesity may encounter weight stigma and perceive it as psychological stress, promoting unhealthy eating behavior.

The bivariate findings indicated that WSSQ was positively correlated with both EE and UE, aligning with the prior literature suggesting that youths who report higher levels of internalized weight stigma may be more likely to exhibit maladaptive eating behaviors. However, because these findings are based on cross-sectional correlations, we cannot infer a causal link between weight stigma and eating behaviors. Interestingly, WSSQ was also positively associated with SCOFF, reflecting the notion that negative weight-related beliefs may contribute to a broader risk for EDs. However, these correlational relationships did not initially extend to significant DBMI-Z, emphasizing the complexity of the pathways linking weight stigma, disordered eating, psychological stress, and weight status [56,57]. These complexities can mask a straightforward relationship between disordered eating patterns and measurable weight change over the time frame of a typical study. Consequently, instruments such as the SCOFF and TFEQ-R21 may not reflect all factors contributing to BMI Z-score outcomes in our GEE model (Table 2).

After excluding non-contributory variables such as the TFEQ subscales and SCOFF (based on QIC considerations), WSSQ became significant, albeit with a modest coefficient. Alternatively, multicollinear variables could have obscured and complicated the direct effect of weight stigma on body weight. These findings highlight the value of model simplification strategies in uncovering subtle effects within interrelated psychosocial constructs [58]. Depending on the final prediction model, youths with higher levels of IWS may be dealing with an elevation of body weight in clinical settings.

Several limitations should be noted in this study. The study sample was relatively predominant in terms of gender (with 63.7% of participants being male), which may limit the generalizability of the findings to female populations. In addition, the reliance on self-report questionnaires may introduce biases such as social desirability and recall inaccuracy of eating behaviors. The HADS is a screening tool and does not assess the clinical diagnosis of anxiety and depression, so we might have a misclassification error in the assessment of these disorders. Experimental works documented the reduction in weight stigma [10,59] and psychological stress [60] via different strategies during the clinical follow-up. Reducing the influence of IWS may reduce clinical attrition and enhance weight loss [22]. However, we did not collect and assess the changes in IWS, psychological stress, eating behaviors, and EDs during the clinical follow-up that determine the effect of the variables. We also did not gather comprehensive sociodemographic information (e.g., place of residence, parents’ educational level, number of family members, or family income), which can play a significant role in obesity management.

Furthermore, this study did not include a community comparison group, limiting our capacity to infer causality or to account for contextual factors that vary across different settings. Taken together, these limitations mean that although we observed an association between weight stigma and maladaptive eating behaviors, we cannot conclude a causal relationship or exclude the influence of unmeasured variables. Future research should consider larger and more diverse samples, incorporate longitudinal or experimental designs, and include relevant sociodemographic data to clarify whether and how weight stigma influences eating patterns and BMI over time.

## 5. Conclusions

In conclusion, this study reinforces the importance of addressing the psychosocial needs of youths with obesity alongside clinical engagement to achieve sustainable improvements in weight trajectories. The findings advocate for an integrative framework that prioritizes psychological well-being and weight stigma reduction in clinical settings. Future research should evaluate interventions aimed at reducing stigma and enhancing psychological resilience in youth with obesity.

## Figures and Tables

**Table 1:** Means, standard deviations, and bivariate correlation among variables (N = 102).

	Mean (SD)	1	2	3	4	5	6	7	8	9	10	11	12	13	14
1. Age	13.22 (2.42)	-													
2. Gender (male)	65 (63.70%)	0.156	-												
3. IBMI	30.83 (4.88)	0.434 **	0.015	-											
4. IBMI-Z	2.14 (0.35)	−0.125	−0.185	0.777 **	-										
5. DBMI-Z	−0.16 (0.51)	0.005	−0.156	0.176	0.178	-									
6. Visit number	7.74 (8.62)	−0.056	0.033	0.049	0.065	−0.428 **	-								
7. Visit interval (years)	0.33 (0.39)	−0.137	−0.212 *	−0.006	0.106	0.078	−0.082	-							
8. WSSQ	33.13 (7.49)	0.045	0.19	0.199 *	0.196 *	0.138	−0.04	−0.061	-						
9. HADS_Anxiety	11.69 (3.69)	−0.155	−0.076	−0.115	0.052	0.135	−0.052	−0.007	−0.147	-					
10. HADS_Depression	8.20 (3.02)	0.175	−0.045	0.106	0.081	−0.116	0.121	0.063	0.137	0.495 **	-				
11. TFEQ-R21_EE	10.93 (4.03)	0.121	0.023	0.030	−0.024	−0.009	0.074	−0.004	0.262 **	−0.104	0.061	-			
12. TFEQ-R21_UE	20.13 (4.96)	−0.280 **	−0.112	0.024	0.239 *	0.051	0.109	0.048	0.323 **	0.099	0.048	0.507 **	-		
13. TFEQ-R21_CR	15.24 (1.62)	0.050	−0.204 *	−0.068	−0.117	−0.008	0.110	−0.124	−0.055	−0.022	0.005	0.018	0.126	-	
14. SCOFF	1.25 (1.46)	0.086	0.120	0.051	0.008	0.095	0.025	0.063	0.426 **	0.195 *	0.269 **	0.115	0.170	−0.150	-
* *p* < 0.05, ** *p* < 0.01															

IBMI: initial body mass index. IBMI-Z: initial body mass index z-scores. DBMI-Z: difference in body mass index z-scores. WSSQ: Weight Self Stigma Questionnaire. HADS: Hospital Anxiety Depression Score. TFEQ-R21: Three-Factor Eating questionnaire in 21 statements. EE: emotional eating. UE: uncontrolled eating. CR: cognitive restraint. SCOFF: Sick, Control, One, Fat, Food questionnaire.

**Table 2:** Baseline predictors and subsequent change in BMI Z-scores.

Variables	Coefficient	95% CI		*p*-Value
		Lower Limit	Upper Limit	
Intercept	−1.232	−2.097	−0.367	0.005
Age	0.016	−0.014	0.046	0.285
Gender	−0.093	−0.237	0.052	0.209
IBMI-Z	1.342	1.127	1.557	<0.001
Visit number	−0.028	−0.039	−0.017	<0.001
Visit interval time	0.008	−0.053	0.069	0.805
WSSQ	0.002	−0.010	0.014	0.762
HADS_Anxiety	0.050	0.026	0.074	<0.001
HADS_Depression	−0.081	−0.099	−0.063	<0.001
TFEQ-R21_EE	0.014	−0.005	0.032	0.149
TFEQ-R21_UE	0.001	−0.021	0.022	0.954
TFEQ-R21_CR	0.019	−0.020	0.057	0.341
SCOFF	0.035	−0.038	0.108	0.346

IBMI-Z: initial body mass index z-scores. WSSQ: Weight Self Stigma Questionnaire. HADS: Hospital Anxiety Depression Score. TFEQ-R21: Three-Factor Eating questionnaire in 21 statements. EE: emotional eating. UE: uncontrolled eating. CR: cognitive restraint. SCOFF: Sick, Control, One, Fat, Food questionnaire.

**Table 3:** Baseline predictors without variables of eating behavior and eating disorders and subsequent change in BMI Z-scores.

Variables	Coefficient	95% CI		*p*-Value
		Lower Limit	Upper Limit	
Intercept	−0.682	−1.189	−0.174	0.008
Age	0.013	−0.002	0.027	0.084
Gender	−0.150	−0.236	−0.064	<0.001
IBMI-Z	1.363	1.244	1.481	<0.001
Visit number	−0.025	−0.029	−0.021	<0.001
Visit interval time	−0.014	−0.103	0.074	0.750
WSSQ	0.006	0.000	0.011	0.034

## Data Availability

The original contributions presented in this study are included in the article material. Further inquiries can be directed to the corresponding author.
